# Serosurveillance of *Orientia tsutsugamushi* and *Rickettsia typhi* in Bangladesh

**DOI:** 10.4269/ajtmh.13-0570

**Published:** 2014-09-03

**Authors:** Rapeephan R. Maude, Richard J. Maude, Aniruddha Ghose, M. Robed Amin, M. Belalul Islam, Mohammad Ali, M. Shafiqul Bari, M. Ishaque Majumder, Ampai Tanganuchitcharnchai, Arjen M. Dondorp, Daniel H. Paris, Robin L. Bailey, M. Abul Faiz, Stuart D. Blacksell, Nicholas P. J. Day

**Affiliations:** Mahidol-Oxford Tropical Medicine Research Unit, Faculty of Tropical Medicine, Mahidol University, Bangkok, Thailand; Centre for Tropical Medicine, Nuffield Department of Clinical Medicine, Churchill Hospital, Oxford, United Kingdom; Chittagong Medical College, Chittagong, Bangladesh; Dhaka Medical College, Dhaka, Bangladesh; Comilla Medical College, Comilla, Bangladesh; Shaheed Ziaur Rahman Medical College, Bogra, Bangladesh; MAG Osmani Medical College, Sylhet, Bangladesh; Sir Salimullah Medical College, Dhaka, Bangladesh; London School of Hygiene and Tropical Medicine, London, United Kingdom

## Abstract

Scrub and murine typhus infections are under-diagnosed causes of febrile illness across the tropics, and it is not known how common they are in Bangladesh. We conducted a prospective seroepidemiologic survey across six major teaching hospitals in Bangladesh by using an IgM enzyme-linked immunosorbent assay. Results indicated recent exposure (287 of 1,209, 23.7% seropositive for *Orientia tsutsugamushi* and 805 of 1,209, 66.6% seropositive for *Rickettsia typhi*). Seropositive rates were different in each region. However, there was no geographic clustering of seropositive results for both organisms. There was no difference between those from rural or urban areas. *Rickettsia typhi* seroreactivity was positively correlated with age. Scrub typhus and murine typhus should be considered as possible causes of infection in Bangladesh.

The etiology of febrile illness remains poorly characterized in many places in the developing world. The causative pathogens are usually not identified and patients are often treated empirically with antimicrobial chemotherapy. Although the burden of some infections is believed to be substantial (e.g. enteric fever), many others, including scrub and murine typhus, are less well described and frequently under-diagnosed.^1^,^2^

Scrub typhus is caused by *Orientia tsutsugamushi*, an obligate intracellular bacterium transmitted by the bite of larval trombiculid mites (chiggers, *Leptotrombidium* spp). This disease is endemic to Asia, the Pacific region, and Australia. In Southeast Asia alone, an estimated one million cases of scrub typhus occur annually and there are 50,000–80,000 deaths per year caused by this disease,^3^ although this number of deaths is probably an underestimate.

Clinical manifestations are non-specific and include fever, headache, myalgia, eschar, and rash. There is little published evidence for the occurrence of scrub typhus in Bangladesh. One case series of 40 rickettsial infection included 24 patients (60%) positive for scrub typhus by using the Weil-Felix test.^4^

Murine typhus is a zoonosis caused by *Rickettsia typhi*, a small, obligate intracellular bacterium transmitted by rat fleas (*Xenopsylla cheopis*).^5^,^6^ It has a worldwide distribution, particularly in tropical climates in areas with large populations of rodents and fleas. Although cases are regularly documented in the United States, Mexico, and Europe, murine typhus is under-recognized in tropical regions.^6^ Only one case series of seven Bangladeshi nationals in Singapore has been reported.^7^

The mainstay of diagnosis for both diseases is serologic analysis, but there is a lack of awareness and access to appropriate diagnostics, which leads to inappropriate treatment, excess morbidity, and a substantial economic impact in the most densely populated regions of the world. Exposure to these infections may be common in Bangladesh because of similar geographic characteristics of this country to parts of the Asia-Pacific region where these organisms are known to be prevalent. A study was performed to determine the IgM seroprevalence as a marker of recent exposure to *O. tsutsugamushi* and *R. typhi* across Bangladesh.

Patients were recruited during June–August 2010 at Chittagong (n = 250), Dhaka (n = 200), Sir Salimullah (Dhaka) (n = 200), Comilla (n = 200), Bogra (n = 200), and Sylhet (n = 200) Medical College Hospitals in Bangladesh. These hospitals are government tertiary care hospitals with large catchment areas^8^ covering four of the seven divisions of Bangladesh. Unselected patients of all ages and both genders who came to a hospital and had a blood test for another purpose were screened for the study. Inclusion criteria were patients providing written informed consent and having sufficient remaining serum or plasma from a blood test taken for another purpose. There were no exclusion criteria. Informed consent was obtained from all adult participants and from the parents or legal guardians of minors. Age, sex, area of residence, and occupation were recorded.

The study was approved by the Bangladesh Medical Research Council Ethics Committee, the London School of Hygiene and Tropical Medicine Ethics Committee, and the Oxford Tropical Research Ethics Committee.

Enzyme-linked immunosorbent assays (ELISAs) were used for detection of human IgM specific for *O. tsutsugamushi* and *R. typhi*. The antigens used in this study were whole cell antigens of the Karp and Gilliam strains of *O. tsutsugamushi* and the Wilmington strain of *R. typhi*. The negative and positive control samples were pooled positive serum samples from two Thai patients with ELISA net optical densities (ODs) < 0.2 and > 1.0, respectively.

The ELISA was adapted from a method described for IgG.^9^ Serum or plasma was diluted 1:100. One hundred microliter samples were transferred to rickettsial antigen–coated, U-bottom, 96-well microtiter plates and incubated at 37°C for 1 hour. The plates were washed with phosphate-buffered saline (Oxoid Ltd., Basingstoke, United Kingdom) containing 0.05% Tween 20. Bound IgM was detected by a 30-minute incubation with anti-human IgM peroxidase (1:3,000 dilution, 100 μL per well; Invitrogen, Carlsbad, CA). The plates were then washed, and 100 μL of tetramethylbenzidine substrate (Kirkegaard and Perry Laboratories, Gaithersburg, MD) was added to each well. The plates were incubated in a dark chamber at room temperature for 30 minutes and 100 μL of 1 M HCl was added to each well.

Plates were read at a wavelength of 450 nm (minus a reference OD value read at 630 nm) with a microtiter plate reader. The ODs from the wells without antigen were subtracted as background absorbance. Positive serum or plasma samples were identified by using a cutoff of the average of two absorbance values (net OD) ≥ 0.2 derived from a previous study in Thailand (Blacksell SD et al., unpublished data). Positive control serum samples were used to assess quality; we had a requirement that the net OD had to be within ± 2 SD of that found in previous assays using the same method and negative serum samples had to have an OD < 0.2. In the absence of a consensus OD cutoff for an IgM ELISA, a conservative cutoff of ≥ 1.0 was also used in the analysis.

Statistical analysis was performed using STATA 11 SE (StataCorp LP, College Station, TX). Univariate group comparisons were performed using chi-square and Fisher's exact tests, and correlations were assessed using Spearman's rank test. The associations of average net OD with age were determined using linear regression with the least squares method. Statistical significance was set at the 5% level.

A total of 1,250 patients were enrolled into the study, of which 41 were excluded because of inadequate specimens for analysis. The median age of patients was 40 years (interquartile range = 26–55 years) and 54.8% (682 of 1,244) were male. Of 1,209 serum samples examined for antibody, we found that when we used a cutoff net OD of 0.2, 23.7% (287 of 1,209) were seropositive for *O. tsutsugamushi*, 66.6% (805 of 1,209) were seropositive for *R. typhi*, and 6.4% (77 of 1,209) were seropositive for both organisms. When we used an OD cutoff of 1.0, these values were 9.0%, 44.9%, and 4.7% respectively. Net OD correlated with age for patients infected with *R. typhi* (*P* < 0.001) but not for patients infected with *O. tsutsugamushi* (*P* = 0.13).

Sex and occupational risk for seropositivity to *O. tsutsugamushi* and *R. typhi* are shown in Tables 1 and 2. Students were found to be less likely than persons with other occupations to be seropositive for antibodies to both organisms at both OD cutoffs. With a cutoff of 1.0 (Table 2), we found that farmers had a reduced risk of exposure to *O. tsutsugamushi* and housewives had an increased risk of exposure to *R. typhi*. There was no clear geographic clustering of seropositive persons by residential address or association with urban versus rural area of residence.

The percentage of seropositive persons from each study hospital is shown in Figure 1. Comilla had the highest seroprevalence for *R. typhi* at both cutoffs. Seroprevalence of *O. tsutsugamushi* was highest in Comilla at a cutoff of 0.2, and seroprevalence of *O. tsutsugamushi* was highest in Chittagong at a cutoff of 1.0.

**Figure 1.**
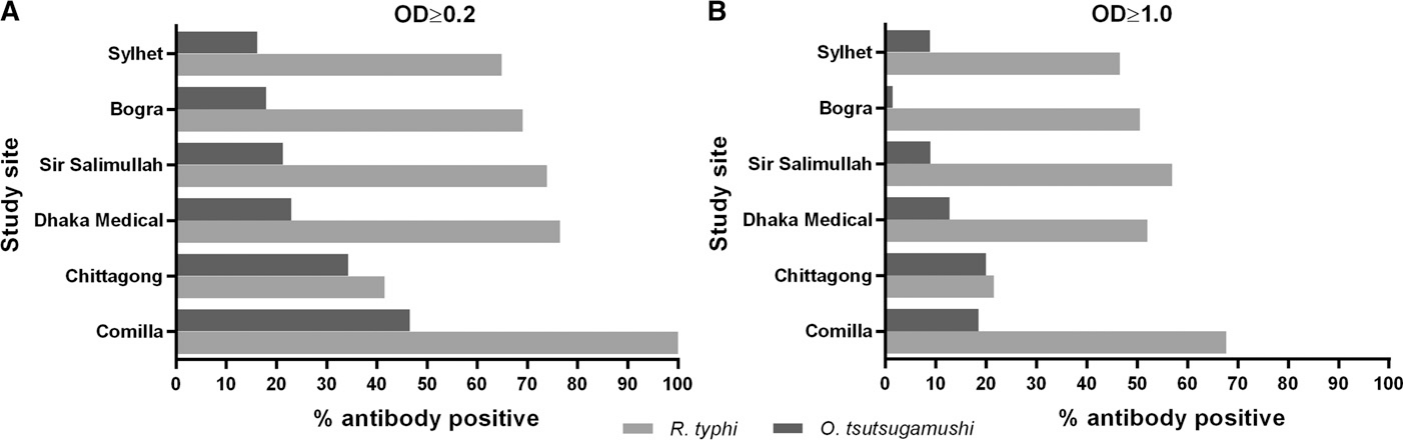
Percent seropositive to *Orientia tsutsugamushi* and *Rickettsia typhi* from each study site, Bangladesh, with optical density (OD) cutoffs of **A,** 0.2 and **B,** 1.0.

Approximately 24% of patients in this study had serologic evidence of exposure to *O. tsutsugamushi*, and almost 67% had antibodies to *R. typhi*. These rates are comparable to those in previous studies in the Asia-Pacific region for scrub typhus but much higher than those for murine typhus.^9^,^10^ This relatively high rate of exposure to *R. typhi* in Bangladesh may be related to poor sanitation and high numbers of rodent hosts.^11^,^12^ Further studies would be required to verify this.

There was a significant correlation of net OD for *R. typhi* with age, similar to that found in Indonesia^9^ where one postulated explanation was differing rates of occupational exposure in different age groups. The present study found students to have lower rates, and housewives a higher rate, of seropositivity to *R. typhi*. There was no association with age for scrub typhus, as found elsewhere.^9^

The primary serologic assay for detection of *O. tsutsugamushi* and *R. typhi* antibodies is indirect immunofluorescence. It was not used in this study because there is a lack of reproducibility and agreement about its interpretation and methods.^10^,^13^ In comparison, the ELISA is more suitable for screening purposes because it is cheaper, more reproducible when using an automated procedure, and can be performed quickly on large numbers of samples. In addition, it is easier to compare ELISA results with those of other studies because it is more suited to seroprevalence surveys of this type.^9^,^14^,^15^

There were some limitations to this study. It used an ELISA for detection of IgM, which is less specific than IgG and persists in the blood for a much shorter period after infection. This indicates recent exposure to the organism, including some acutely infected persons, but limited comparison with results of other studies which used detection of IgG. It did not include any assessment of clinical disease thus inferences could only be made about prevalence of exposure to the organisms. No information could be obtained on the infecting strain of *Rickettsia*. It is possible that different undetected strains are prevalent in Bangladesh and we are thus underestimating seropositivity. Conversely, cross-reactivity with antibody against other organisms may have led to an overestimation of exposure for the two organisms studied.

In light of these results, scrub and murine typhus should be considered as possible causes of febrile illness in Bangladesh. Future studies will be necessary to investigate which strains of scrub and murine typhus are present in Bangladesh in vectors, reservoirs, and humans, as well as to determine the incidence and spectrum of clinical disease.

## Figures and Tables

**Table 1:** Sex and commonest occupations with risk of seropositivity with an optical density cutoff of 0.2 for *Orientia tsutsugamushi* and *Rickettsia typhi* in Bangladesh*

Variable	All patients, n = 1,244		*Orientia tsutsugamushi*, n = 287					*Rickettsia typhi*, n = 805				
	No.	%	No.	% Positive	*P*	Risk ratio	95% CI	No.	% Positive	*P*	Risk ratio	95% CI
Male	682	54.8	152	23.1	0.55	0.9	0.8–1.1	440	66.9%	0.78	1.0	0.9–1.1
Housewife	452	37.5	117	26.5	0.59	1.1	0.9–1.3	303	68.6%	0.26	1.0	1.0–1.1
Farmer	185	15.4	41	22.9	0.37	0.9	0.7–1.2	121	71.5%	0.13	1.1	1.0–1.2
Service	183	15.2	43	24.2	0.64	0.9	0.7–1.2	121	68.0%	0.66	1.0	0.9–1.1
Student	148	12.3	19	13.3	**0.0003**	0.5	0.3–0.7	71	49.7%	**< 0.0001**	0.7	0.6–0.9
Businessman	102	8.5	25	25.5	1.0	1.0	0.7–1.4	62	63.3%	0.48	0.9	0.8–1.1

* Values in bold are statistically significant. CI = confidence interval.

**Table 2:** Sex and commonest occupations with risk of seropositivity with an optical density cutoff of 1.0 for *Orientia tsutsugamushi* and *Rickettsia typhi* in Bangladesh*

Variable	All patients, n = 1,244		*Orientia tsutsugamushi*, n = 109					*Rickettsia typhi*, n = 543				
	No.	%	No.	% Positive	*P*	Risk ratio	95% CI	No.	% Positive	*P*	Risk ratio	95% CI
Male	682	54.8	58	8.8	0.77	0.9	0.7–1.4	284	43.2	0.18	0.9	0.8–1.0
Housewife	452	37.5	43	9.7	0.07	0.7	0.5–1.0	224	50.7	**0.02**	1.2	1.0–1.3
Farmer	185	15.4	12	6.7	**0.02**	0.5	0.3–0.9	84	46.9	0.81	1.0	0.9–1.2
Service	183	15.2	19	10.7	0.57	0.9	0.6–1.4	79	44.4	0.62	1.0	0.8–1.1
Student	148	12.3	9	6.3	**0.03**	0.5	0.3–0.9	39	27.3	**< 0.0001**	0.6	0.4–0.7
Businessman	102	8.5	13	13.3	0.68	1.1	0.7–1.9	38	38.8	0.13	0.8	0.6–1.1

* Values in bold are statistically significant. CI = confidence interval.
